# Perspectives of youth in Ireland on school-based mental health and suicide prevention: the MYSTORY study

**DOI:** 10.1093/heapro/daad049

**Published:** 2023-06-03

**Authors:** Eibhlin H Walsh, Matthew P Herring, Jennifer McMahon

**Affiliations:** School, Child & Youth (SCY) Mental Health and Wellbeing Research Lab, National Institute of Studies in Education, Health Research Institute, University of Limerick, Limerick, Ireland; Department of Psychology, University of Limerick, Limerick, Ireland; Physical Activity for Health Cluster, Health Research Institute, University of Limerick, Limerick, Ireland; Department of Physical Education and Sports Sciences, University of Limerick, Limerick, Ireland; School, Child & Youth (SCY) Mental Health and Wellbeing Research Lab, National Institute of Studies in Education, Health Research Institute, University of Limerick, Limerick, Ireland; Department of Psychology, University of Limerick, Limerick, Ireland

**Keywords:** school mental health promotion, school-based suicide prevention, youth voice, participatory research

## Abstract

Anxiety, depression, and suicide are leading causes of disability and death among young people, globally. Schools are an ideal setting to target young people’s mental health, yet young people’s beliefs about and experiences with school mental health and suicide prevention are not well understood. This gap in knowledge contradicts both national and international youth mental health recommendations and the United Nations Convention on the Rights of the Child, which collectively advocate for understanding young people’s perspectives on matters concerning them, including school mental health. Therefore, the Mental Health of Youth Story (MYSTORY) study explored young people’s perspectives on school mental health and suicide prevention using a participatory-based approach incorporating photovoice. MYSTORY consisted of a community/university partnership involving young people as participants (*n* = 14) and advisors (*n* = 6). Experiential, reflexive thematic analysis (TA) within a critical approach generated three themes relating to young people’s experiences with and beliefs about school mental health promotion and suicide prevention. Findings highlight the critical role of schools in impacting young people’s mental health, with the need to amplify youth voice and involvement in school mental health evident. Our study addresses an important gap by employing participatory-based approaches to explore young people’s perspectives on school mental health and suicide prevention. This is the first known study to explore young people’s perspectives on their voice and involvement in school mental health. Findings have important implications for youth and school mental health and suicide prevention research, policy, and practice.

## INTRODUCTION

Anxiety, depression, and suicide are leading causes of disability and death among adolescents, globally (World Health Organization [WHO], 2021a, 2021b). Suicide has risen among young people over the last decade (Centers for Disease Control and Prevention, 2020; Office for National Statistics, 2020). Young people’s mental health has deteriorated during COVID-19 (Samji *et al.*, 2022) and is now even more of a critical societal concern.

Schools are an ideal setting to promote young people’s mental health, given schools’ capacity to: (i) reach diverse young people, (ii) facilitate social connections, and (iii) host wide-ranging interventions (Ross *et al.*, 2017; Marinucci *et al.*, 2022). School-based mental health promotion encompasses a continuum of interventions, including social and emotional learning, life skills promotion, and emotional and behavioural issues prevention, all of which are enhanced by involving key stakeholders, such as young people (Weist and Murray, 2008). School-based suicide prevention involves targeting suicidal thoughts and behaviours as primary intervention outcomes and indirectly through wellbeing outcomes and can include curriculum-based and gatekeeper-focussed interventions, as well as crisis support (Miller, 2011; Walsh *et al.*, 2022). Post-primary, school-based mental health promotion and suicide prevention interventions have demonstrated effectiveness (Feiss *et al.*, 2019; Walsh *et al.*, 2022), with positive impacts far-reaching to communities (Weare and Nind, 2011).

Despite the clear benefits of targeting young people’s mental health in school settings, young people’s beliefs about and experiences with school-based mental health promotion and suicide prevention are not well understood. In Canada and Australia young people were dissatisfied with the scarcity of mental health supports in school (Bowers *et al.*, 2013; Marinucci *et al.*, 2022). Young people engaging with school-based prevention supports in the United States (US) reported: (i) exclusion from decision-making, (ii) positive and negative perceptions of risk assessments, and (iii) traumatic experiences with external interventions (Kodish *et al.*, 2020). Despite Ireland’s school mental health policy advocating for an enhanced understanding of young people’s perspectives on school mental health (Department of Education and Skills [DES], 2019), there is little known about young people’s perspectives on school mental health and suicide prevention in Ireland.

The importance of understanding young people’s perspectives on school mental health and suicide prevention is two-fold. Firstly, young people’s views are key determinants of school mental health intervention effectiveness, with a review identifying adolescents’ perspectives on school mental health interventions as a key predictor of postvention mental health outcomes (Rojas-Andrade and Bahamondes, 2019). Secondly, Article 12 of the United Nations (UN) Convention on the Rights of the Child (UNCRC) mandates that young people have a right to express their views on matters affecting them (UN Human Rights, 1990). Too often young people’s knowledge is assigned lesser value in comparison to adult-centred knowledge (Chimirri, 2019). As such, research exploring young people’s perspectives should be informed by youth-centred paradigms, such as the advocacy/participatory worldview, which prioritizes inquiry ‘with’ young people, as opposed to ‘on’ young people, while striving for positive changes in young people’s lives [Kemmis and Wilkinson (1998), as cited by Creswell and Poth (Creswell and Poth, 2016)]. Participatory research approaches aim to engage participants more equitably in research, by striving to increase agency and mitigate issues of power (Water 2018; Teixeira *et al.*, 2021).

Research should meaningfully involve young people using methods which align with young people’s developmental stage, particularly when sensitive topics are being explored (such as mental health), which are often associated with more challenges (Dickson-Swift *et al.*, 2007). A population-based study in Australia demonstrated that only 16.4% of participants were classified as having adequate mental health literacy levels (Lam, 2014), and a study in Ireland involving 187 participants indicated that adolescents had difficulties with aspects of mental health literacy, including identifying depression and potential suicidality (Byrne *et al.*, 2015). Photovoice is a rigorous, participatory-based research method which integrates empowerment education, feminist theory, and documentary photography, to represent participants’ needs, encourage action, and inform policy (Wang and Burris, 1994). Photovoice has shown particular value in exploring young people’s perspectives on and experiences with mental health in an empowering and youth-centred manner, leading to critical thinking and self-reflection (Vélez-Grau, 2019). However, no known study exploring young people’s perspectives on school-based mental health promotion and suicide prevention has used photovoice.

To address the important gaps discussed, the Mental Health of Youth Story (MYSTORY) study aims to explore young people’s experiences with and beliefs about school mental health promotion and suicide prevention, using photovoice.

## METHODOLOGY

We took a critical view to using the Consolidated Criteria for Qualitative Research (COREQ) (Tong *et al.*, 2007) to inform our inquiry, as some COREQ components did not align with our analytical approach, including: (i) data saturation, which is incompatible with reflexive thematic analysis (TA) (Braun and Clarke, 2019b) and (ii) participant checking, given the researchers' role in data interpretation and due to participants not remaining in the same place in time throughout the research process (Braun and Clarke, 2021b).

### Theoretical framework and reflexivity

The critical paradigm reflects our assumption that individuals exist within power-laden and inequitable environments, which means that power is always at play (Leavy, 2017). Arguably young people are marginalized by power dynamics between themselves and adults (Facca *et al.*, 2020; Pincock and Jones, 2020), which can manifest as perceptions (both implicit and explicit) that young people are incapable of making informed decisions about matters impacting them (Bradbury-Jones *et al.*, 2018). Settings for young people, such as school, contain significant power imbalances between adults and young people [Robinson and Kellett (2004), as cited by Gallagher (Gallagher, 2008)]. Moreover, Ireland is a cultural context which does not have a notable history of student voice (Skerritt *et al.*, 2021b). As such, power is a particularly important concept to consider when exploring experiences with and perspectives related to school mental health and suicide prevention with young people in Ireland.

MYSTORY is underpinned by contextualist and critical approaches to epistemology. In line with Guba and Lincoln (Guba and Lincoln, 1994), we believe that reality is shaped by social, political, cultural, economic, ethnic and gender values. Research from a critical perspective strives to target issues in social life, such as social justice and marginalism while considering issues of power in the research process. There are inevitable power imbalances between participants and researchers (Scotland, 2012; Leavy, 2017), insofar that researchers determine research questions, methods, and outputs. Participatory research both acknowledges power as a central research component and offers a way to intervene with unavoidable notions of power, by placing enhanced value on young people’s authentic knowledge and perspectives on the world. Furthermore, participatory methods are arguably more ethically acceptable research methods, due to their alignment with the UNCRC (Gallagher, 2008). Ultimately, we generated knowledge through the interaction between data and our own perspectives, research interests, and experiences (Madill *et al.*, 2000; Scotland, 2012). Therefore, knowledge generated through inquiry is socially constructed, situationally dependent, and impacted by power differentials (Madill *et al.*, 2000; Brendel and Jäger, 2005; Scotland, 2012), which leads to value-mediated findings (Guba and Lincoln, 1994).

The first author (EHW) is a female doctoral student, with experience in mentoring young people in a national voluntary organization. The supervising author (JMM) has expertise in engaging young people in mental health projects. EHW and JMM engaged with young people involved in MYSTORY and engaged in critical reflexivity (Spencer *et al.*, 2020), by reflecting on and discussing their influence on the research. EHW used a research diary to enhance reflexivity by: (i) reflecting on the influence of identity and personal insights (Rabiee, 2004; Leavy, 2017; Meskell *et al.*, 2021), (ii) appraising how actions and relationships could influence young people’s engagement (Berger, 2015; Water, 2018; Montreuil *et al.*, 2021; Abma *et al.*, 2022), and (iii) reflecting on sensitive aspects of MYSTORY (Rodriguez, 2018) and the unprecedented complexities related to COVID-19.

### MYSTORY

MYSTORY consisted of a university-community partnership between the University of Limerick and a community, youth mental health organization based in the mid-west of Ireland from October 2020 to 2022, which will be referred to as our community partner herein. A male, youth mental health worker working as a youth advocate with our community partner facilitated the young people’s engagement with MYSTORY, and he will be referred to as the MYSTORY gatekeeper herein. The MYSTORY gatekeeper had an existing professional but trusted and friendly relationship with the young people involved in MYSTORY and had no vested interest in the research outcomes. The MYSTORY gatekeeper’s ‘insider’ positioning was beneficial to the young people involved in MYSTORY, in the context of the previously discussed unavoidable power differentials between participants and researchers. Furthermore, having a dedicated staff member is frequently cited as a key facilitator to young people’s involvement in health-related research (Sellars *et al.*, 2020). The MYSTORY gatekeeper facilitated engagement in MYSTORY by: (i) assisting with the recruitment of participants and the young person advisory group (YPAG), (ii) advocating on behalf of the young people involved in MYSTORY, and (iii) supporting the young people when they encountered any issues with MYSTORY, including during COVID-19 restrictions.

The wider aims of MYSTORY were to: (1) explore young people’s perspectives on youth and school mental health and suicide prevention and (ii) communicate key insights on youth and school mental health and suicide prevention to schools, communities, and the wider public. MYSTORY incorporated photovoice workshops, focus groups, and a photovoice exhibition. Only as far as the first focus groups will be discussed in the present manuscript (Supplementary Figure 1 outlines the MYSTORY timeline). a separate manuscript focussing on the wider MYSTORY study is in preparation.

### Participants, research advisors, and recruitment

Our community partner worked with two groups of young people, a senior group comprised of 19- to 24-year-olds and a junior group comprised of 16- to 19-year-olds, who both advised on service development and mental health advocacy. Young people involved in these groups engaged with MYSTORY as research advisors (as part of a YPAG) and as participants. The YPAG consisted of six young adults who were part of our community partner’s senior group. We collaborated with the YPAG over four meetings to: (i) springboard ideas, (ii) gain greater insight into young people’s experiences, and (iii) gain perspective on aspects of MYSTORY, including the workshops, photography theme and focus group question development. Fifteen participants aged 16–19 years from our community partner’s junior group were purposively sampled (all but one participant took part in the focus group). In the focus group, there were 8 females, 5 males and one individual who did not disclose their gender. All but three participants were attending mainstream post-primary school at the time of data collection, with the remaining three participants recently exiting post-primary school. An electronic information sheet provided participants and their parents/guardians with both information on and ethical considerations to MYSTORY. We obtained consent/assent through email. EHW rang parents/guardians of participants aged under 18 years of age to confirm their participation and to address any questions about MYSTORY. We also consulted with the participants themselves to tailor aspects of MYSTORY, where possible. We evaluate MYSTORY as *Youth consulted and informed,* based on Roger Hart’s Ladder of Young People’s Participation (Funk *et al.*, 2012).

### Procedure

Focus groups with young people can be challenging, with young people’s developmental stage making them more susceptible to peer influence, discomfort, and shyness (Norris *et al.*, 2012). However, photovoice provides an accessible method for young people to reflect on and express their thoughts and feelings through capturing and sharing photos (Wang, 2006) and enables discussions rooted in the participants’ lives (Wang and Burris, 1994; Hergenrather *et al.*, 2009; O’Latz, 2017). Photovoice is also useful for exploring sensitive topics with young people (Wang, 2006), which can be challenging to discuss in traditional focus groups (Wellings *et al.*, 2000; Punch, 2002). Exploring school mental health promotion and suicide prevention ‘with’ young people who have contextualized their unique experiences using photovoice is well situated in our theoretical framework, which seeks to explore the unique and contextually-specific experiences of young people in our sample (Creswell and Poth, 2016), while acknowledging our influence on the study findings.

Two preparatory photovoice workshops featuring an audio-visual presentation were facilitated by EHW, JMM, the MYSTORY gatekeeper and a professional photographer (who was also a YPAG member) on Microsoft Teams, for the participants to: (i) discuss MYSTORY, (ii) build rapport and co-design ground rules, and (iii) develop skills needed to engage in MYSTORY (i.e. photography skills and ethical considerations, in line with O’Latz, 2017). Workshops were interactive, developmentally appropriate, and goal-oriented, similar to previous procedures (Vélez-Grau, 2019; Pincock and Jones, 2020).

During workshop 1 participants were tasked with taking one photo to engage in photo elicitation at workshop 2 and during workshop 2 participants were instructed to capture three photos for the focus group, representing personal, community, and school mental health. A protocol outlining non-appropriate content for photos (i.e. violence and discrimination) was shared with the participants in the workshops and through email.

We developed scripted photo prompts and open-ended focus group questions informed by the YPAG. Two focus groups containing seven participants each were conducted. Photo elicitation was interwoven throughout the focus group discussions on school mental health and suicide prevention, with participants sharing the meaning of one of their photos. Focus group questions were non-leading and aimed to understand experiences (i.e. What are your thoughts on school mental health and suicide prevention?) and beliefs (i.e. ‘If you had a magic wand what would you do for mental health and suicide prevention in school?’). To support participants’ potential distress during the focus groups, a mental health professional was available to provide support.

### Analytical approach

Our ontological and epistemological stance guided the use of experiential, reflexive TA, to explore experiences and beliefs regarding school-based mental health and suicide prevention (Braun and Clarke, 2021a; Wiltshire and Ronkainen, 2021). EHW led data analysis. Firstly, EHW transcribed the data, which aided data familiarization. Data familiarization was further enhanced by reading the transcribed data and listening to the audio in an iterative manner, while noting patterns and observations. Then, data was coded using NVivo with respect to the research questions, grouping excerpts into codes to form initial themes. EHW and JMM discussed the generated codes and initial themes, in the context of: (i) their own experiences of engaging with young people both generally and during MYSTORY, and (ii) the theoretical and conceptual underpinnings of MYSTORY, including the concept of power between young people and adults. This collaboration led to theme refinement and finalization (Braun and Clarke, 2020). Semantic, latent, deductive and inductive themes were generated from this process (Braun and Clarke, 2019a). Participant excerpts have been assigned non-identifiable codes. Minor edits were made to excerpts to hide identifiable information and for clarification which is denoted by ‘[..]’.

## RESULTS

Photography topics included school mental health, mental health difficulties and mental health promotion (Supplementary Figures 2, 3 and 4 are example photos). Three themes relating to school mental health and suicide prevention were generated (Supplementary Table 1 outlines the coding table and includes exemplar quotations).

### Aspects of schooling experiences unhelpful to young people’s mental health

Many participants believed that school-based mental health supports, such as counselling, mental health awareness and identification of mental health difficulties could be potentially a huge benefit to young people. Moreover, there was a sense of expectation that schools should address young people’s mental health difficulties and needs, given the significant portion of time that young people spend in school. However, participants believed that key aspects of their schooling experiences, such as academics, social and school mental health, were unhelpful to young people’s mental health.

Participants believed that the impact of academics on young people’s mental health was two-fold. Firstly, academic achievement pressures could lead to mental health difficulties among young people, with exam pressures and peer comparisons of academics associated with psychological distress, feelings of inadequacy and concerns about the future after post-primary school. Some participants believed that feelings of inadequacy bridged challenges with academics and mental health, as indicated in the following excerpt:

It just creates a thing like, that just because you can’t do this thing to the extent that everyone else can you’re not like, as good, you’re not like.. it kind of takes a dig at yourself (KH814).

Secondly, academics were described as indirectly impacting on the accessibility of school mental health supports. School mental health supports, such as mental health workshops and programmes, were ringfenced for non-exam years. Some participants also expressed the perception that school personnel were dissatisfied when students were engaging in non-academic activities, due to concerns that students would miss out on academics. Some participants who had experienced mental health difficulties shared the belief that there was no room in the classroom for mental health difficulties, with school-based supports mostly inaccessible until such difficulties were noticeable in the classroom. Furthermore, some participants engaging with school mental health supports shared a sense of dismay due to having to take ownership in coordinating their supports, which is challenging in the busy school environment. The following excerpt captures one participant’s experience of having a panic attack in school:

Having a panic attack in school and because I had it there they had to, they had to, they had no choice but to like, give me support and like, talk to me and see if I was okay and then going back into school this year that same level of support just wasn’t there [..] unless you are literally crying in the classroom or crying to the guidance counsellor they don’t really take you seriously (KH814).

Moreover, some participants believed that social aspects of their schooling experiences were unhelpful to young people’s mental health. Mental health difficulties coming to the fore in the classroom was described as embarrassing, due to peers’ observations of these difficulties. Although not limited to the school environment, participants expressed how challenges with sharing mental health difficulties with peers and friends were unhelpful to their mental health. Furthermore, conformity and hiding aspects of oneself were also noted as social aspects of school which could exacerbate mental health difficulties. One participant shared their experiences of mental health difficulties in the context of school through both their photography and their discussions in the focus group, as captured in the following excerpt:

This is my photo, it’s the daffodils in my garden and I made sure to include the wall behind them cos of.. in the sense of.. it’s based on school and feeling trapped, cos everything is really structured, and how some people can flourish in that and there’s no problem but some people kind of break in that and wilt and it’s not for everyone.. [..]. For me and my own self-harm, I know that it was kind of a thing that school contributed to, it wasn’t the main factor, it was a contributory factor definitely, it feels like there is no control over your own self and identity because you do have to dress like this, you need to do this right in this right way (UX851).

Finally, some participants’ experiences with school mental health supports were perceived to be largely unhelpful to their mental health. Firstly, some participants described awareness campaigns, one-on-one interventions (such as mindfulness) and classroom-based activities as unrelatable and ‘childish’. Secondly, mental health workshops and awareness days (even those perceived favourably) were described as unhelpful due to their infrequency, with participants expressing the importance of these initiatives on a regular basis throughout their entire years of schooling.

### School personnel as ‘frontline’ for supporting young people with their mental health: a double-edged sword.

Participants believed that school personnel were ideal candidates to identify and assist young people with their mental health. Despite the expectation that school personnel (including teachers and counsellors) should be frontline in supporting young people with their mental health, perceived structural, interpersonal, and professional barriers were described to hinder support.

Perceived kindness, empathy and availability were described as key characteristics of supportive school personnel (including teachers, counsellors, coaches, and principals). Many participants described the importance of being treated with kindness and feeling understood when engaging with school personnel who support young people with their mental health. Some participants indicated their preference for support from younger school personnel, due to the perception that younger school personnel are more understanding of the issues that can impact young people’s mental health today. Moreover, the knowledge that participants could reach out to a supportive school staff member at any time during school if needed, was key to feeling supported with their mental health in school. What was integral to this perception was the informal relationships between young people and school personnel supporting young people with their mental health, particularly with reference to teachers whom some participants described as a type of friend.

Many participants identified guidance counsellors as key school personnel to support young people with their mental health, with most participants who received school-based mental health support engaging with counsellors. Participants identified teachers as having the potential to be particularly important in supporting young people with their mental health, given teachers’ enhanced capacity for both proximal and emotional closeness with young people, in comparison to counsellors whom young people more seldomly interact with. The following excerpt illustrates one participant’s positive experience of being supported by their guidance counsellor, who was also their teacher:

When I was in third year I had really like, bad anxiety [..] I don’t know if it was because my guidance counsellor was my teacher as well in school [..] so I’m not sure if that had anything got to do with him being extra supportive [..] but he said I could come to him any time and I had appointments with him every week, and am, he was really helpful and he really helped me when I was under a lot of stress (HD567).

In contrast, some participants expressed dismay with school personnel being perceived as key individuals to support young people with their mental health, due to perceived structural, interpersonal, and professional barriers hindering support. Many participants believed that teachers, principals, and guidance counsellors had multiple responsibilities in their roles, which impinged their capacity to support young people with their mental health. Participants felt that there were not enough school personnel dedicated to supporting young people and that there was a need for school personnel who had the designated and sole responsibility of supporting young people with their mental health. Although guidance counsellors were described to most commonly support young people with their mental health, guidance counsellors were also subject teachers for some participants. Moreover guidance counsellors were largely perceived to provide guidance concerning professional development in comparison to mental health support. Furthermore, many participants stated that there were not enough guidance counsellors available to support young people with their mental health. Moreover, school personnel were perceived as not always having the capacity to support young people with their mental health. Teachers were described as having the potential to impact young people’s wellbeing both positively and negatively, given the important role that teachers have in young people’s lives. Some participants felt that teachers who were dismissive of mental health should not be tasked with supporting young people. Moreover, some participants who were receiving school-based support felt that their mental health difficulties were too complex to be addressed adequately in school and were referred to their general practitioner. One participant who was experiencing suicidal ideation shared their discontentment with their external referral for support:

Instead of sending me to the counsellor or anything like that they just told me to go to the general practitioner, and, am, I basically just had to, basically kind of forced to say ‘oh I am fine’ or whatever, something like that, to my GP, like everything with me is okay and that I could go back to school (YC512).

### The need for young people’s involvement and (typically unheard) voice in school mental health

Participants perceived young people’s involvement in school mental health as integral for effectively targeting young people’s mental health. However, a lack of young people’s involvement and voice in key pillars of school mental health, such as school mental health initiatives (i.e. mental health awareness days), school-based interventions (i.e. peer-delivered programmes) and student consultation spaces was central to participants’ experiences. Participants felt that the lack of their voice extended to wider aspects of school.

Many participants felt that young people mostly passively received school mental health supports which lacked consultation with young people, from one-to-one supports (including mindfulness interventions) to group approaches (including class-based interventions). Participants believed that sharing young people’s lived experience with mental health could enhance the relevancy of supports. Furthermore, participants recognized the need for school mental health initiatives, including mental health programmes, which align with the unique contextual needs of schools, given the diversity of schools’ needs and issues. Participants believed that diverse young people’s voices informing approaches to mental health in school were needed to ensure that supports could be helpful. One participant shared their belief about the importance of involving young people in the process of identifying what targets are needed for school mental health and suicide prevention given the diversity of schools’ needs, in the following excerpt:

Even if any programme came in [to the school] and we had a focus group like we’re doing now about the issues in that particular school and what could be targeted I think that would do a lot more good than a one size fits all approach to their programmes [..] we all have different issues in a mixed school, all-girls schools, all-boys schools, so I think that the issues are so different and how people deal with the issues are so different, that dealing with individual student bodies, rather than you know people sitting in a room deciding this is the programme were rolling out and going with that in every school, it won’t have the same effect as ‘what are your issues and how can we help?’ and work on that and then go back to the school with a sort of tailored approach. (UX851).

Peer-delivered school mental health programmes were one type of intervention that some participants felt would actively involve young people. Having young people involved in peer-delivered school mental health programmes may circumvent generational differences between young people and adults, which could lead to young people feeling more at ease and understood in sharing their experiences with peer helpers. Two of the participants who engaged in peer-delivered interventions shared their positive experiences with and beliefs about these interventions, with benefits described for both themselves as peer helpers and for the peers engaging with the intervention as a recipient. Recipients were perceived to develop confidence and have improved mental health through sharing their experiences, while peer helpers developed greater mental health literacy and skills through supporting peers. Conversely, participants did have concerns that peer helpers could become overwhelmed and may not be confidential in their role. One of the participants described the peer-delivered programme they took part in through discussion of their experiences and visually through their photograph:

When I was in transition year I was part of this group we used to call peer education and we, am, got trained on how to like, say for our school we did like, we presented a PowerPoint to help people come to us, if they wanted to talk to someone we could direct them to a counsellor or something [..] When we were in peer education we learned about the mental health balance. There is going to be things in your life that negatively impact your mental health, but it is very important that you have positive impacts that balance out so you’re not weighed down by the negative. (HD567)

Moreover, there was a shared sense of apprehension that enhancing young people’s voice and involvement in school mental health may not result in meaningful change, with participants alluding to young people’s lack of agency in the school environment. Participants shared the need to strengthen links between young people and adults in the wider school context, for productive (and much-needed) student-school personnel collaboration in mental health initiatives. Suggestions included involving impartial adults (i.e. non-school personnel) in such collaborations, harnessing mutual respect between school personnel and young people, and student anonymity when providing feedback to school personnel.

Finally, many participants recognized that meaningful involvement and voice lacked in existing student consultation spaces in schools, such as student councils. Participants (both representatives and non-representatives of student councils) believed that student councils did not harness agency to make meaningful and youth-led changes. One participant described adult selection of student council representatives in their school. Another participant who was a student council representative highlighted the lack of opportunities to get to know and consult with students in their school.

## DISCUSSION

We explored young people’s experiences with and beliefs about school mental health promotion and suicide prevention, using reflexive TA within a critical approach. Our findings support schools as a key setting for supporting young people, with school environments, personnel and supports identified as considerably impacting on young people’s mental health, in both positive and negative ways. Our findings contribute to the understanding of young people’s perspectives on their voice and involvement in school mental health in the wider literature and are among the first of their kind in the Irish context. Methodologically, our research addresses a clear gap in emancipatory-focussed, participatory-based studies exploring young people’s perspectives on school mental health and suicide prevention.

Broadly, our findings align with international research investigating schooling experience determinants of young people’s mental health. Firstly, findings from 13 countries similarly show that academic-related pressures undermine the positive aspects of the school environment (i.e. provisions of support, learning, safety and socialization) on mental health (Johns Hopkins Bloomberg School of Public Health and UN Children’s Fund, 2022). Secondly, young people’s difficulties accessing school mental health supports was also reported in Canada and Australia (Bowers *et al.*, 2013; Marinucci *et al.*, 2022). Thirdly, young people in Canada and the UK similarly perceived teachers to have limited capacity to support young people’s mental health (Bowers *et al.*, 2013; O’ Reilly *et al.*, 2018). Fourthly, students in Thailand also perceived teachers to play a key role in suicide prevention (Chaniang *et al.*, 2022). Finally, research conducted in the UK, Scandinavia, and Australia similarly reported perceived barriers of structural, interpersonal and professional nature hindering young people’s attainment of support from school personnel (O’ Reilly *et al.*, 2018; Ekornes, 2020; Kostenius *et al.*, 2020; Marinucci *et al.*, 2022).

Young people in our study identified school personnel as having the potential to play a key role in supporting young people with their mental health, which aligns with research demonstrating that young people in Australia had a preference for teachers in a supportive capacity (Marinucci *et al.*, 2022) and were less likely to seek out formal mental health supports, including mental health professionals or general practitioners (Grové and Marinucci, 2022). Furthermore, our finding that young people were unsatisfied with referral to external mental health supports resonates with findings that: (i) at-risk young people in the US negatively perceived external interventions, administered with little consultation with young people (Kodish *et al.*, 2020), and (ii) that school’s referral of at-risk young people to external supports led to decreases in help-seeking behaviours, due to young people’s desire for support from school personnel (Evans and Hurrell, 2016). Young people’s experiences of external referral in our study align with school mental health policy in Ireland, which directs schools to refer at-risk students to local health services or general practitioners (National Educational Psychological Service, 2016).

Our findings draw attention to young people’s awareness of their lack of and desire for young people’s voice and meaningful involvement in school mental health interventions and student consultation spaces. Previous research also demonstrates that young people perceive youth voice as central to school mental health (Hennessey *et al.*, 2022), but schools do not consult with young people to understand their perspectives on school mental health (Marinucci *et al.*, 2022). What is unique to our study is the perceptions that: (i) mental health interventions and consultation spaces lack and need meaningful involvement and voice of young people, and (ii) young people’s involvement and voice may not result in meaningful change in school mental health, given their lack of agency in the school environment. These insights align with the critical perspectives’ assumption that individuals are impacted in their daily lives by power inequalities (Leavy, 2017), which are likely more evasive for young people in school settings, given the unavoidable imbalances between school personnel and students (Fielding, 2004). We contextualize our findings in terms of ‘adultism’, which has an underlying assumption that adults are more knowledgeable about issues relevant to young people, leading to decision-making by adults on youth-centred issues (Teixeira *et al.*, 2021). Issues of adultism likely extend to the wider school setting. Despite the introduction of student councils in Irish post-primary schools in 1992 as a forum for addressing matters concerning youth, in response to the UN Charter on the Rights of the Child, it has been argued that student councils have become tokenistic and exclusionary through a lack of consultative youth voice (Fleming, 2015), which is further evidenced by our findings.

Notably, young people in our study recognized the need for diverse youth voice in identifying and improving school mental health and suicide prevention, which aligns with emerging recommendations for enhancing school mental health through the employment of interventions which target socio-cultural elements of school life (Jessiman *et al.*, 2022). Moreover, participants identified peer supports as a key way to support young people’s mental health in school, which aligns with findings among Thai adolescents demonstrating that peer supports were considered a key suicide prevention strategy (Chaniang *et al.*, 2022). In our study, central to the positive perceptions of peer-delivered interventions was that peers are accessible and relevant to young people. Similarly, young people perceived peers as more understanding when providing mental health support (Johns Hopkins Bloomberg School of Public Health and UN Children’s Fund, 2022). Despite peer-led mental health interventions having the potential to target various mental health outcomes (i.e. suicidal behaviours and depression), and showing evidence of positive development in peer leaders, evidence of intervention effectiveness is lacking (King and Fazel, 2021).

### METHOD AND DATA REFLECTIONS

Given that participants were involved in an advisory capacity in a mental health organization, our sample may have had greater mental health literacy and interest in comparison to that of the general population. Purposive sampling of young people involved in mental health organizations has been previously used in qualitative mental health research (Mawn *et al.*, 2016). Furthermore, our sample size of 14 participants is similar to other school mental health-related studies using focus group methodologies (Marinucci *et al.*, 2022) and incorporating photovoice (Hergenrather *et al.*, 2009; Vélez-Grau, 2019). Our sample size aligns with our aims to maintain particularization rather than aiming for generalization of contextualized samples (Kaluzeviciute *et al.*, 2022).

It is important to consider the potential impact of COVID-19 restrictions on our data. A notetaker is recommended in focus group research to record non-verbal interactions (Rabiee, 2004; Hergenrather and Rhodes, 2008), however, this was not possible in our study due to COVID-19 restrictions. During the focus groups face masks may have hindered non-verbal communication, which may have further impacted the degree to which participants felt comfortable interacting with one another, a fundamental aspect to focus group methodology (Tong *et al.*, 2007; Hergenrather and Rhodes, 2008). Given the rich generated themes in our study, we believe the rapport and trust developed during MYSTORY mitigated the potential negative impacts of COVID-19 restrictions on our findings.

Research locates children’s voices within adult-designed frameworks of power, including participatory methods (Spencer *et al.*, 2020). A difficulty in research exploring sensitive topics is the need for adult-driven decision-making, such as the confidentiality breach incorporated in MYSTORY, which was informed by standard procedures in child protection (Department of Children and Youth Affairs, 2017). Despite the unavoidable impact of power in our research, we believe that our rigorous methodology ensures that power biases were kept to a minimum. Similar to previous studies, our study design was constrained within parameters governed by (lack of) funding (Pincock and Jones, 2020; Luescher *et al.*, 2021), resulting in aspects of MYSTORY which could not pragmatically involve young people.

#### Implications

The parallels of our findings demonstrating the impact of school on young people’s mental health with research conducted in various cultural contexts, warrant international-wide responses in research, policy, and practice which strive to ensure that schools help (and not hinder) young people’s mental health.

Internationally, there are calls for decision-makers to centre mental health and wellbeing efforts on the voices of young people (Lancet, 2022). Realizing the potential of school mental health and suicide prevention will not be possible without youth voice. A lack of youth voice in the school settings contrasts with the mandates of Article 12 of the UNCRC, and therefore more research is needed to further investigate the extent to which young people are involved in school mental health and suicide prevention. The participatory approaches employed in MYSTORY may offer a model for engaging young people to understand their unique perspectives on and experiences with school mental health and suicide prevention. Given the likelihood that student voice policies will considerably vary in both content and enactment across different types of schools (Skerritt *et al.*, 2021a), we urge decision-makers to advocate for youth voice in school mental health policy in ways which recognizes schools’ needs.

Ireland’s policy for enhancing school mental health aims to improve key areas by 2023, including *Relationships and Partnerships,* which further aims to have suitably qualified school personnel to support young people with personal and social needs and crises (DES, 2019). Beyond Ireland, the US Healthy People 2030 goals aim to increase the number of adolescents who have an adult available to support them (Office of Disease Prevention and Health Promotion, 2022). As such, our findings that young people would like more school personnel designated to support them with their mental health, in the context of National and International youth wellbeing policy aims, strengthens the need to increase the accessibility and availability of school personnel who can support young people with their mental health. Schools under the directive of policy mandating that at-risk young people are referred on to external supports should ensure that young people receive a transparent explanation of this process and why it is in place, to alleviate unmet expectations of young people relating to follow-up supports for at-risk students. If there was scope to alter existing processes surrounding external supports referral based on youth voice, schools could incorporate *NICE guidelines for the management of self-harm in the UK* into policy focussing on intervening with at-risk students. NICE guidelines advocate for the important role of school personnel and are evidence-based guidelines informed by young people’s voices (The Association for Child and Adolescent Mental Health, 2022).

We recommend that researchers consult with young people to enhance research relevancy and acceptability. International calls are increasing to involve young people as co-researchers and place greater value on young people’s perspectives in research (Schelbe *et al.*, 2015; MacSweeney *et al.*, 2019). We recommend involving young people in research, in ways which are stimulating, empowering and beneficial for young people, through participatory research methods. However, we do recognize the challenges to involving young people in research, with less than 1% of health-related studies concerning young people including young people’s input (Sellars *et al.*, 2021). Greater avenues for funding and resources are needed to support young people’s meaningful involvement in the research process.

We recommend the following to school decision-makers based on our findings: (i) mental health promotion should be should evident across schooling years; (ii) the links between academics and young people’s mental health need to be carefully considered; (iii) facilitate rapport building opportunities for young people and school personnel; (iv) greater accessibility of school personnel to support youth mental health; (v) consult with young people so that they can fully understand the extent to which schools can provide support (particularly where suicidal thoughts and behaviours are concerned); and (vi) involve young people in school mental health and suicide prevention, from identifying critical intervention targets specific to their school’s needs, to delivering evidence-based interventions to peers.

## CONCLUSION

Our findings address important and timely gaps in understanding young people’s experience with and beliefs about school mental health promotion and suicide prevention. Schools have the potential to be a key setting to support young people with their mental health, but school mental health promotion and suicide prevention must be shaped by youth voice and the specific needs of school contexts must be given due consideration in the scaffolding of school mental health supports. Our findings are relevant to researchers in the field of school mental health, and more broadly interested in youth wellbeing, youth public health and education.

## Supplementary Material

daad049_suppl_Supplementary_Figure_S1

daad049_suppl_Supplementary_Figure_S2

daad049_suppl_Supplementary_Figure_S3

daad049_suppl_Supplementary_Figure_S4

daad049_suppl_Supplementary_Table_S1
