# Supplementary figures and images for: Perspectives of youth in Ireland on school-based mental health and suicide prevention: the MYSTORY study
Source: Health Promot Int. 2023 Jun 3;38(3):daad049. doi: 10.1093/heapro/daad049 (PMC10243762; doi:10.1093/heapro/daad049)

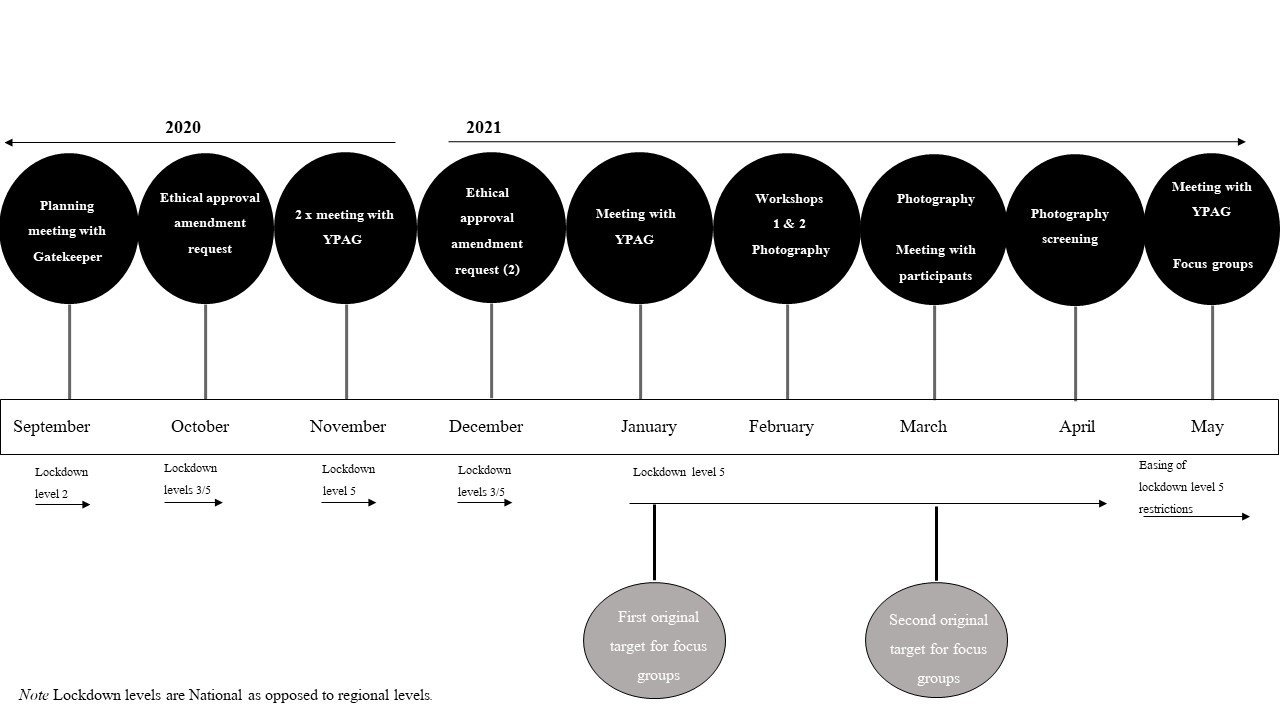

Supplement: daad049_suppl_Supplementary_Figure_S1 [file daad049_suppl_supplementary_figure_s1.jpeg]

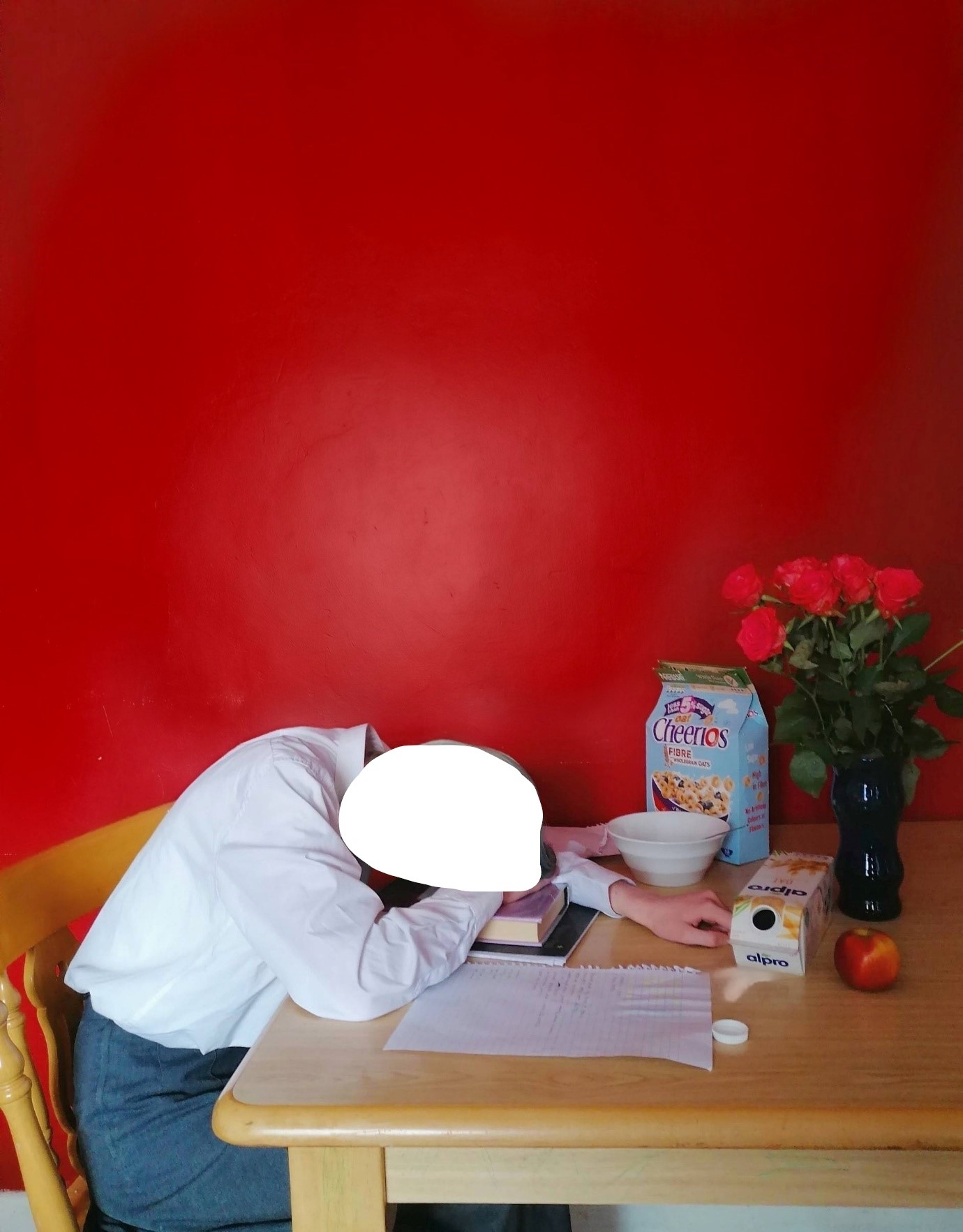

Supplement: daad049_suppl_Supplementary_Figure_S2 [file daad049_suppl_supplementary_figure_s2.jpeg]

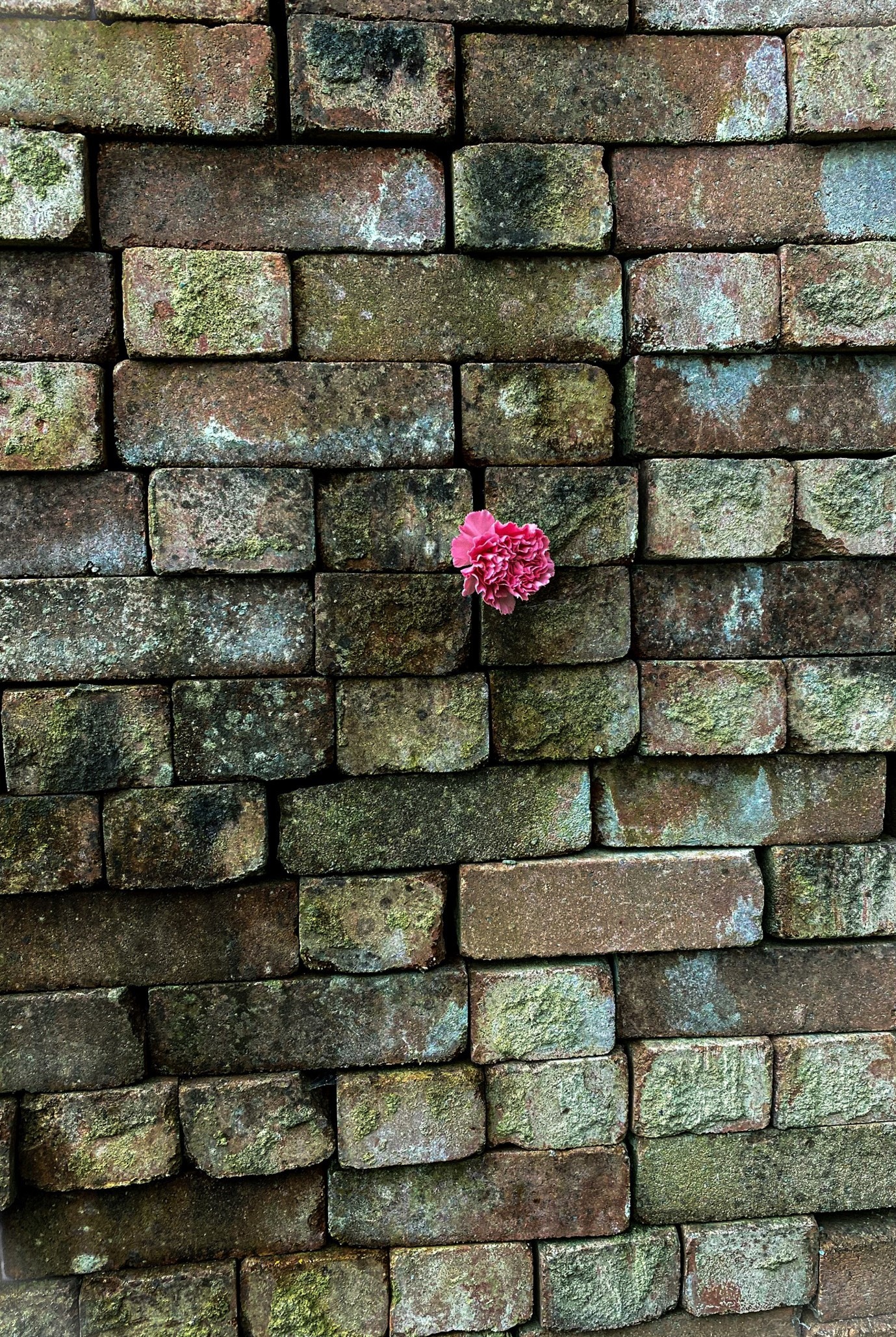

Supplement: daad049_suppl_Supplementary_Figure_S3 [file daad049_suppl_supplementary_figure_s3.jpeg]

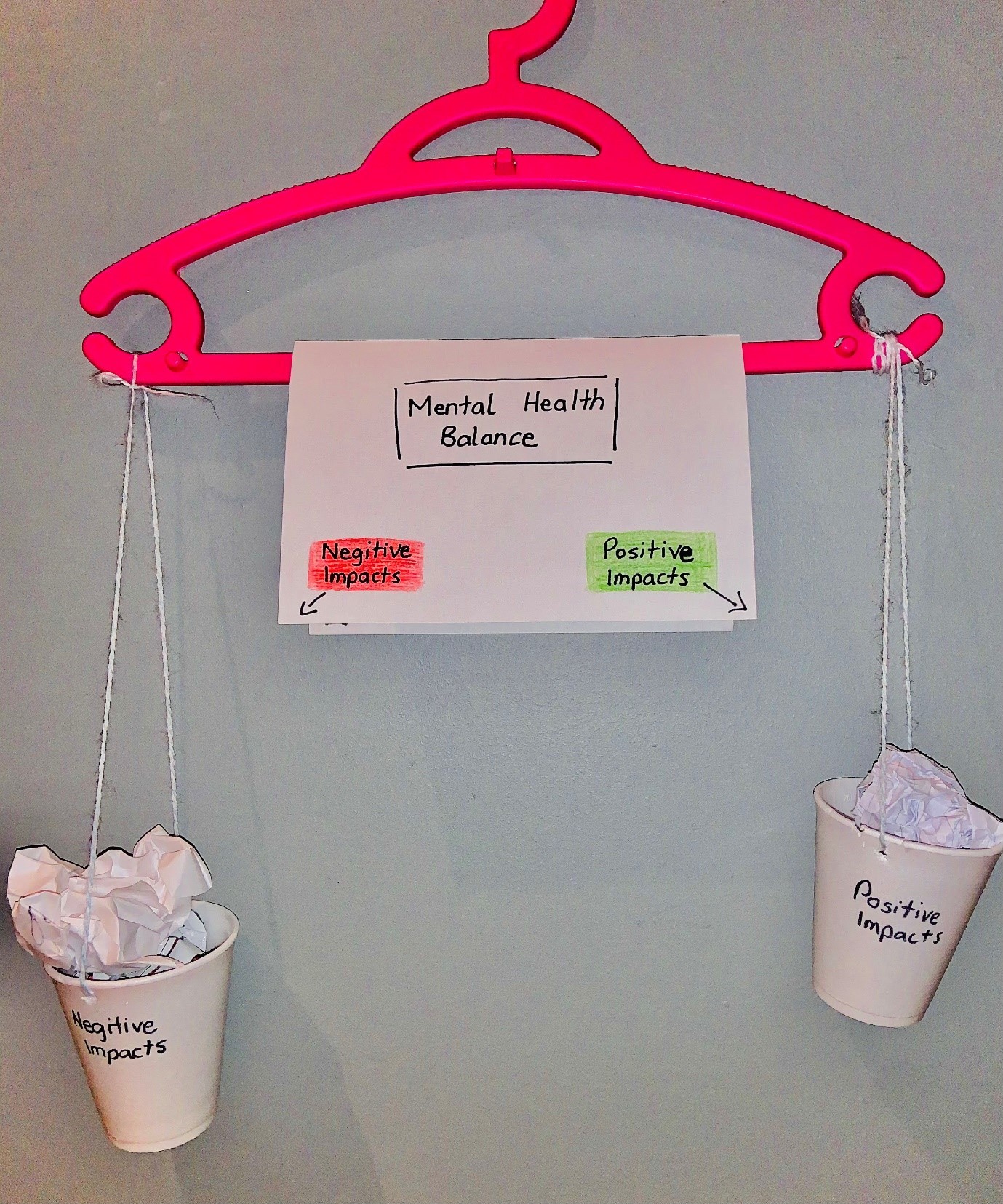

Supplement: daad049_suppl_Supplementary_Figure_S4 [file daad049_suppl_supplementary_figure_s4.jpeg]
