# Supplementary material for: Perspectives of youth in Ireland on school-based mental health and suicide prevention: the MYSTORY study
Source: Health Promot Int. 2023 Jun 3;38(3):daad049. doi: 10.1093/heapro/daad049 (PMC10243762; doi:10.1093/heapro/daad049)
Supplement: daad049_suppl_Supplementary_Table_S1 [file daad049_suppl_supplementary_table_s1.docx]

**Supplementary Table 1: Coding table of themes, codes, and exemplar quotations**

| **Theme name** | **Codes** | **Exemplar quotations** |
| --- | --- | --- |
| **Aspects of schooling experiences unhelpful to young people’s mental health** | Academics as of utmost importance in schools  Academics linked to mental health difficulties  Failure to attain academic achievement hinders progression to the next stages of life  Academic achievement as an indicator of value in people  Self-worth linking academic achievement difficulties and mental health difficulties  Greater availability of mental health-related workshops during non-academic years  School environment as trapping  Little space to experience mental health difficulties in the classroom  Feeling alone when dealing with mental health difficulties  Having to hide aspects of identity in the school environment  Unwanted attention from peers when mental health difficulties are noticeable in the classroom  Greater availability of support in school when mental health begins to impact the school environment  Lack of sustained initiatives which positively impact young people’s mental health  Positive experiences with school based mental health programmes  School mental health supports as unrelatable and unhelpful  Onus on young people to co-ordinate their school-based mental health supports  Value in identifying mental health difficulties in schools  Expectation of schools to support young people with their mental health needs  Lack of trust in schools to host mental health supports for young people | That’s all its’ [school] focused on like, just grades (WB763)  I think a lot of the problems that people can encounter in school can sort of start around basing your self-worth off of what you produce, the work that you do and the results you get (UX851)  I had really like, bad anxiety and the whole exam stress on top of it as well was just not helping (HD567)  The positive affirmations isn’t going to get rid of my thoughts or like, get rid of having panic attacks or anxiety attacks, or like anything like that, it’s just like a positive affirmation, it’s not like, anything to actually like, to do with how you should talk to people, could talk to people, how you can respond to people if they are talking to you about their mental health [..] it’s never like actually about mental health or like, how to cope with everything, and it’s just like, it just seems really like false, it’s like, it’s just like tick a box (KH814)  We did like, a load of workshops in transition year I feel like that was the only time our school kind of does things like that is with the transition year students who don’t have to focus on exams (GV703)  There is 600 students in my school alone and every single one of them could be suffering with something completely different, and if there was something that big to help them in school where they go, five days a week for 8 hours it could be perfect, it could be amazing, it could be life-changing for them (LT763) |
| **School personnel as “frontline" for supporting young people with their mental health: a double-edged sword** | Guidance counsellors as key to supporting young people with their mental health in school  Guidance counsellors provide career support instead of mental health support  Guidance counsellors having lack of competency to support young people with their mental health  Not enough guidance counsellors to provide mental health support  Positive impact of teachers on young people’s mental health  Negative impact of teachers on young people’s mental health  Teachers as a type of friend  Dislike of confiding in teachers perceived as unfriendly  Dismissal of mental health by teachers  Teachers expected to be a key supportive adult in supporting young people with their mental health  Teachers as among the closest adults to young people  Teachers having the potential to be key in identifying mental health needs in young people  Importance of perceiving school personnel as available to provide support  Importance of kindness in school personnel when supporting young people  Importance of feeling that school personnel could understand what young people experience with their mental health  Greater understanding of young people’s mental health by younger school personnel  Importance of informal relationship between young people and school personnel who support young people with their mental health  School personnel not always having the time or capacity to support young people with their mental health  Need for school personnel who solely provide mental health support  Referral of mental health difficulties to external supports  Preference to receive mental health support from school personnel  The importance of everyday interactions with school personnel | Because they [guidance counsellors] are guiding you with your life but they’re not really guiding you with your mental health most of the time (PQ471)  Have more guidance counsellors available because sometimes one guidance counsellor isn’t enough for like, 600 students you know? One person isn't realistically capable of looking after like, or listening to 600 people, you know? it’s not realistic (HD567)  Our vice principal, she was really nice and I think she did help a lot of people but she was very busy, but she helped people whenever she could. There was a time where she helped me when I was in distress, and she’s young and she has kids of her own she has been through like the stuff that we have been through so she was very understanding and like, empathetic (CG742)  Like they [teacher] clearly not like, don’t have an education around mental health (KH814)  They’re [teachers] are kind of like frontline I suppose they are probably the closest people to students, like they maybe talk to you most as well every day, they probably see things more than anyone else would, even more than the guidance counsellor probably (WB763)  If they were there all day every day in their office and like, their only responsibility was us and our mental health rather than being a teacher of a subject at the same time (DL091) |
| **The need for young people’s involvement and (typically unheard) voice in school mental health** | Young people are not taken seriously both inside and outside of school  Young people as passive receivers of school mental health supports  Desire for school mental health interventions informed by young people  One size fits all approaches to school mental health programmes  Need for tailored and youth-informed school mental health approaches  Diversity as important in young people’s involvement in school mental health  Different schools have different issues  Inability of student voice to make changes  Importance of young people’s voice in school mental health  Lack of agency of young people  Lack of agency for student council members to connect with young people  Adult-led identification of students for student councils  Discontentment with student councils due to lack of youth-led change  Preference for peer-delivered school mental health programmes due to enhanced relatability to young people  Benefits for young people receiving peer-delivered programmes  Benefits for young people delivering peer-delivered programmes  Concern that peer helpers may not act in confidence in their role  Concern that peer helpers may become overwhelmed in their role  Importance of mutual respect between young people and school personnel collaborating in school mental health  Need for anonymous communication between young people and school personnel | I think that with youth things in schools and or even outside of schools, sometimes they’re not taken seriously because they’re the youth [..]…I think sometimes it’s just like "they don’t know what they are talking about, their just like, the students" when really there wouldn’t be a school without the students, it wouldn’t make sense (KH814)  when we have this whole concept of a student group, helping people and like, feeling like your voices are heard or feeling like something is happening, a big drawback is you are trying to help but you are not actually able to change anything for them if that makes sense (PQ471)  I think having something from people in the school from students that are maybe experiencing the same things that you are in the same environment, that could be a lot more beneficial than even I think programmes [..] I think having that kind of insight into the school environment and what can be done on that level, because every school is so different, could be very beneficial for students if it's done well (UX851)  Young people know young people’s issues (JU678)  I was a member of the student council and they’re kind of like, supposed to help students, students didn’t really come up to us and we weren’t really given the chance to talk to students, tell them like "oh if you want to talk to us you can", we just kind of had a little badge, but we never got to like communicate, apart from like the tea club we did last time, we got talk to students and they got to see who we really were, I feel like they didn’t really know, if that makes sense (GV703)  We have like, student council.. that’s kind of it, we have like clubs whatever, but they're not.. they haven’t changed anything since I’ve gotten here, they haven’t changed anything since first year, every single year people roll up and nothing happens, I feel like with something like this it would be incredibly important to have something actually happening (PQ471)  I do think having some kind of programme where students are trained in how to deal with people coming to them, and be like “look, you know this is how I am feeling” and kind of not being judgemental or taken aback by that, and sort of trying to understand, you know, what they are going through (UX851)  I think that it was good that they got it, for us [peer helper] to deliver to other years because am, they might be able to relate to a young person, rather than an older person (HD567) |
